# Disrupt of Intra-Limb APA Pattern in Parkinsonian Patients Performing Index-Finger Flexion

**DOI:** 10.3389/fphys.2018.01745

**Published:** 2018-12-03

**Authors:** Francesco Bolzoni, Roberto Esposti, Silvia M. Marchese, Nicoló G. Pozzi, Uri E. Ramirez-Pasos, Ioannis U. Isaias, Paolo Cavallari

**Affiliations:** ^1^Human Physiology Section of the Department of Pathophysiology and Transplantation, Università degli Studi di Milano, Milan, Italy; ^2^Department of Neurology, University Hospital and Julius Maximilians University Würzburg, Würzburg, Germany

**Keywords:** intra-limb anticipatory postural adjustments, Parkinson disease, basal ganglia, motor control, human

## Abstract

Voluntary movements induce postural perturbations which are counteracted by anticipatory postural adjustments (APAs). These actions are known to build up long fixation chains toward available support points (*inter-limb* APAs), so as to grant whole body equilibrium. Moreover, recent studies highlighted that APAs also build-up short fixation chains, within the same limb where a distal segment is moved (*intra-limb* APAs), aimed at stabilizing the proximal segments. The neural structures generating intra-limb APAs still need investigations; the present study aims to compare focal movement kinematics and intra-limb APA latencies and pattern between healthy subjects and parkinsonian patients, assuming the latter as a model of basal ganglia dysfunction. Intra-limb APAs that stabilize the arm when the index-finger is briskly flexed were recorded in 13 parkinsonian patients and in 10 age-matched healthy subjects. Index-finger movement was smaller in parkinsonian patients vs. healthy subjects (*p* = 0.01) and more delayed with respect to the onset of the prime mover flexor digitorum superficialis (FDS, *p* < 0.0001). In agreement with the literature, in all healthy subjects the FDS activation was preceded by an inhibitory intra-limb APA in biceps brachii (BB) and anterior deltoid (AD), and almost simultaneous to an excitatory intra-limb APA in triceps brachii (TB). In parkinsonian patients, no significant differences were found for TB and AD intra-limb APA timings, however only four patients showed an inhibitory intra-limb APA in BB, while other four did not show any BB intra-limb APAs and five actually developed a BB excitation. The frequency of occurrence of *normal sign*, *lacking*, and *inverted* BB APAs was different in healthy vs. parkinsonian participants (*p* = 0.0016). The observed alterations in index-finger kinematics and intra-limb APA pattern in parkinsonian patients suggest that basal ganglia, in addition to shaping the focal movement, may also contribute to intra-limb APA control.

## Introduction

Anticipatory postural adjustments (APAs) represent a crucial aspect of the voluntary movement organization. Throughout their feed-forward control, APAs are able to limit the displacement of the center of mass (CoM), caused by the interaction forces induced by the voluntary movement. Indeed, such activities build up fixation chains toward the available support point, where to discharge the interaction forces produced by the voluntary movement, in this way granting the whole body equilibrium (Massion, 1992; Bouisset and Do, 2008). Since these activities usually involve several trunk and limb muscles, they may also be referred to as *inter-limb* APAs (see Cavallari et al., 2016 for a review). However, it has been demonstrated that also movements involving very tiny masses, like an index-finger flexion, are accompanied by APAs (Caronni and Cavallari, 2009). In this case, indeed, specific APAs were observed in arm and shoulder muscles that stabilize the *segmental equilibrium* of the upper limb and optimize the movement performance (Bruttini et al., 2016). Because of their localization with respect to the moving segment, these postural activities were named *intra-limb* APAs (see also Aoki, 1991; Caronni and Cavallari, 2009).

Intra- and inter-limb APAs share not only their principal behavioral features, like the flexibility to adapt to the available support points (Cordo and Nashner, 1982; Bruttini et al., 2014) as well as to the direction and speed of the focal movement (Horak et al., 1984; Aruin and Latash, 1995; Caronni and Cavallari, 2009; Esposti et al., 2015), but also many of the neural structures involved in their control, including primary motor cortex, supplementary motor area, sensorimotor areas (Viallet et al., 1992; Schmitz et al., 2005; Petersen et al., 2009; Ng et al., 2013; Bolzoni et al., 2015). In this regard, some studies correlated neurological diseases with APAs modifications. These experiments not only deepened the knowledge of these pathologies, but also elucidated the structures involved in APAs control. So far, the majority of those studies investigated the effects of pathologies of the central nervous system, like stroke and cerebellar lesions, on inter-limb APAs and on whole-body postural control (Diener et al., 1992; Rajachandrakumar et al., 2016), but the effects of cerebellar lesions was also documented on intra-limb APAs by Bruttini et al. (2015) who reported a disruption of the temporal organization of such postural adjustments. Another subcortical structure that plays a role in movement control is composed of the basal ganglia, and also in this case some studies showed that basal ganglia pathologies correlate with impairments in inter-limb APA control (Viallet et al., 1987; Lee et al., 1995). Since a linkage between basal ganglia and *intra-limb* APAs is still missing, the present study aims to compare the kinematics parameters of the index-finger flexion and the intra-limb APA latencies and pattern between healthy subjects and patients affected by Parkinson disease (PD), assuming the latter as a model of basal ganglia dysfunction.

Considering the well-known role of basal ganglia in shaping the pattern of motor activities driving voluntary movement, one would mainly expect a pattern disruption (i.e., changes in intra-limb APAs sign, excitatory or inhibitory), possibly even associated to a timing alteration.

## Materials and Methods

Thirteen patients affected by PD (PARKINSON group, mean age 60.8 years ± 9.3 SD, four females) and 10 age-matched healthy subjects (HEALTHY group, mean age 61.4 years ± 6.7 SD, six females) were enrolled in this study. Healthy subjects had no history of orthopedic or neurological disorders.

Individual demographic and clinical parameters of PD patients are reported in Table 1. They had no history of orthopedic disorders and followed pharmacological treatments. However, at the time of the experiment, they were in pharmacological wash-out from at least 36 h.

**Table 1:** Demographic and clinical parameters of PD patients.

PARKINSON patient	Age (years)	Disease duration (years)	LEDD (mg)	UPDRS-III total (units)	UPDRS-III upper-limb (units)
1	64	10	610	28	7
2	73	8	560	30	5
3	53	4	640	13	5
4	54	4	555	26	5
5	73	8	1245	11	3
6	76	9	1180	17	2
7	62	9	772	16	5
8	49	11	910	28	7
9	53	4	560	19	5
10	62	12	994	31	6
11	57	6	455	14	5
12	49	3	540	7	2
13	66	5	340	11	2

mean ± SD	60.8 ± 9.3	7.1 ± 3.0	720 ± 280	19.3 ± 8.3	4.5 ± 1.8


*The Levodopa Equivalent Daily Dose of the pharmacological treatment (LEDD) was determined according to Tomlinson et al. (2010). Patients’ evaluation with the Unified Parkinson Disease Rating Scale motor part (UPDRS-III, cfr. Movement Disorder Society Task Force on Rating Scales for Parkinson’s Disease, 2003) is also provided, both as total score and as the dominant upper limb sub-score*.

All participants gave written consent to the procedure, after being informed about the nature of the experiment. The experiments were conducted in conformance with the policies and principles contained in the Declaration of Helsinki and were approved by the Ethical Committee of the University of Milan (counsel 5/16 – 15.02.16).

### Experimental Design

Participants were tested on the dominant limb; the assessment of the handedness was performed according to Oldfield (1971). Participants were sitting and explicitly asked to keep their back supported, the upper-limb still and both feet on the ground. The non-dominant arm was supported by an armrest while the dominant arm was kept along the body, with the elbow flexed at 90°. The hand was prone, in axis with the forearm, with the index-finger pointing forward (i.e., 180° at the metacarpophalangeal joint) while all the other fingers were hanging freely. Subjects kept the back leaning against the seatback and the feet on the ground. The body position was visually checked by the investigator throughout the experiment.

After an acoustic signal, delivered every 7 s, subjects had to perform a self-paced brisk flexion of the index-finger at the metacarpophalangeal joint. Subjects were specifically instructed to perform the movement at will, so as to exclude any reaction-time effect. Each subject performed 45 movements, divided in three sessions of 15 movements with 5–7 min interval in between, in order to avoid fatigue.

### Movement and EMG Recordings

The excursion of the metacarpophalangeal joint was recorded by a strain-gauge goniometer (model F35, Biometrics Ltd^®^, Newport, United Kingdom), fixed with surgical tape. Angular displacement was amplified by a bridge amplifier (model P122, Grass Technologies^®^, West Warwick, RI, United States), which gain was calibrated before each experiment.

Electromyographic (EMG) signals were recorded from the prime mover flexor digitorum superficialis (FDS) and from the biceps brachii (BB), triceps brachii (TB), and anterior deltoid (AD) muscles, involved in the upper-limb postural stabilization (Caronni and Cavallari, 2009). After scrubbing the skin with cotton and alcohol, two pre-gelled surface electrodes (model H124SG, Kendall ARBO, Tyco Healthcare, Neustadt/Donau, Germany) were placed on each muscle, 24 mm apart. Electrode placement for BB, TB, and AD muscles followed the SENIAM guidelines (Freriks and Hermens, 1999). For FDS, SENIAM did not provide specific guidelines; however, the same general approach was adopted: the subject kept the arm and forearm in the experimental position and was asked to repeatedly strongly flex one finger at a time, at the metacarpophalangeal joint. Meanwhile, the experimenter palpated his forearm, so as to isolate the belly of the FDS from that of the surrounding muscles. Electrodes were then placed on the FDS belly, at about 1/3 of the distance of the wrist from the cubital fossa. The selectivity of the EMG recordings was verified by checking that activity from the recorded muscle, during its phasic contraction, was not contaminated by signals from other sources. The EMG signals were amplified (gain 2–10 k) and band-pass filtered (30–1,000 Hz, to minimize both movement artifacts and high-frequency noise) by four differential amplifiers (model IP511, Grass Technologies^®^, West Warwick, RI, United States).

Conditioned goniometric and EMG analog signals were then sampled at 2 kHz with 12-bit resolution by an A/D board (model PCI-6024E, National Instruments^®^, Austin, TX, United States), visualized online and stored for further analysis.

### Data Analysis

Each EMG recording was digitally rectified and integrated (time constant: 10 ms). The onsets of FDS EMG were extracted by running a 1-s mobile-window algorithm over the recording, searching for those positions in which the samples in the 50 ms following the window were all above the mean value +2 SD of the samples within the window. Whenever this criterion was met, the end of the window was considered an onset; all onsets were visually validated. In each muscle, all the 45 EMG recordings were then time aligned to the FDS onset and averaged, so as to obtain an average trace extending from -2000 to +300 ms from the FDS onset, which was then considered time 0; the same was done for the 45 goniometric traces. All subsequent measurements were taken on the averaged traces.

The onset of index-finger flexion was identified on the averaged goniometric trace by applying the same mobile-window algorithm used for FDS onset, but searching for the window position in which all samples in the 50 ms following the window were all below the mean value -2 SD of the samples within the window. Movement amplitude and duration were then measured, respectively, as the amplitude and timing difference between peak index-finger flexion and movement onset. The mean values and variability of the movement latency, amplitude and duration were compared between PARKINSON and HEALTHY groups by means of unpaired *t*-tests and Levene’s tests, respectively. Whenever Levene’s test was significant, the *t*-test for the corresponding variable was corrected for unequal variances estimates.

The onset of an excitatory or inhibitory APA in each postural muscle was searched for on the averaged trace by applying the same moving-window algorithm used for FDS onset; however, the search was stopped at the movement onset, in order to avoid any effect due to re-afferentation triggered by the focal movement. In case an onset was found, if the samples in the 50 ms following the window were all above the mean value + 2 SD of those within the window, the APA was recognized as excitatory, while if the samples in the 50 ms were all below the mean value -2 SD the APA was recognized as inhibitory. If the above criteria failed to identify any onset, it was concluded that the APA was lacking for that muscle. The mean values and variability of the APA latencies, for each postural muscle, were compared between PARKINSON and HEALTHY groups by means of unpaired *t*-tests and Levene’s tests, respectively. Data from patients in which the APA was lacking or had an inverted sign (e.g., excitatory instead of inhibitory) with respect to that observed in healthy subjects (in which APAs always have the same sign, see Results), were excluded from the comparisons.

The pattern of APAs for each postural muscle was assessed in each group by counting the number of participants that showed an inhibitory, excitatory, or lacking intra-limb APA. The frequency of occurrence of the three above outcomes was then compared in the PARKINSON vs. HEALTHY group by the Freeman-Halton extension (2 groups × 3 categories) of the non-parametric Fisher Exact test.

For the sake of completeness, as secondary measurements, linear correlations were tested between the intra-limb APA latencies in the PARKINSON group and each of the following demographic and clinical parameters: patient’s age, disease duration, Levodopa Equivalent Daily Dose of the pharmacological treatment (Tomlinson et al., 2010) and Unified PD Rating Scale motor part (UPDRS-III, cfr. Movement Disorder Society Task Force on Rating Scales for Parkinson’s Disease, 2003; both total score and upper-limb sub-score). Data from patients in which APA was lacking or inverted were excluded also from these analyses. Non-parametric Spearman’s R correlation was evaluated between the sign of intra-limb APAs (-1 when inhibitory, +1 when excitatory, and 0 when lacking) and those same parameters.

For all tests, statistical significance was set at *p* < 0.05. All relevant data for the statistical analyses drawn in this study are included in the manuscript, either in Figure 2 or in Tables 1, 2.

**Table 2:** Individual values of movement amplitude and duration in the HEALTHY and PARKINSON groups, together with the corresponding mean value ± SE.

HEALTHY subject	Movement amplitude (°)	Movement duration (ms)	PARKINSON patient	Movement amplitude (°)	Movement duration (ms)
1	81.8	222	1	31.5	64
2	87.9	220	2	50.2	96
3	83.7	177	3	49.3	115
4	69.4	220	4	34.8	284
5	42.7	144	5	38.6	177
6	68.2	208	6	36.1	253
7	86.7	216	7	67.2	196
8	90.2	209	8	52.2	255
9	53.5	63	9	47.0	174
10	58.5	196	10	55.4	138
			11	33.4	115
			12	38.1	150
			13	34.3	196

mean ± SE	72.2^∗^ ± 5.2	187.5 ± 15.8	mean ± SE	43.7^∗^ ± 3.0	170.2 ± 18.4


*Significant differences between the two groups are marked by ^∗^*.

## Results

**FIGURE 1:**
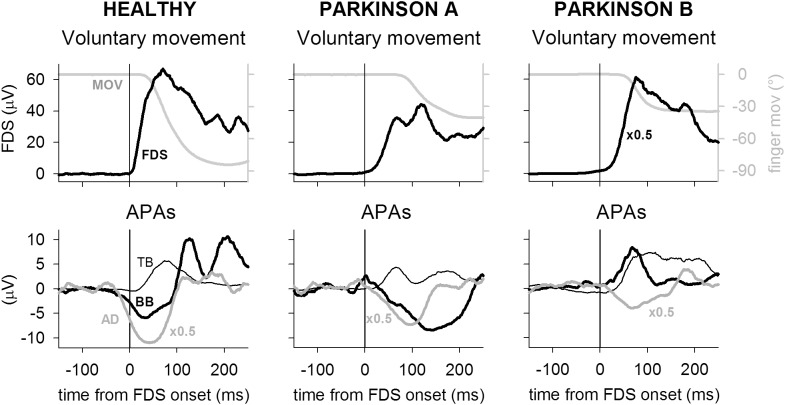
Recordings from one representative participant of the healthy group (HEALTHY) are compared to recordings of two PD patients (PARKINSON A and B). Each trace is the average of 45 recordings, time aligned on the onset of the prime mover flexor digitorum superficialis (FDS). In each participant, top panel shows the FDS activation, matched to the ensuing finger flexion (MOV); bottom panel illustrates the intra-limb APAs in TB, BB, and AD. For illustration purpose, the mean reference signal level, recorded from 750 to 250 ms before FDS onset, has been subtracted from each EMG trace. FDS amplitude has been down-scaled by a factor 2 in patient B and AD amplitude has been down-scaled by a factor 2 in all participants. Note that in the healthy participant the FDS activation was shortly followed by index-finger flexion but preceded by inhibitory intra-limb APAs in BB and AD and almost synchronous with an excitatory intra-limb APA in TB. In both PD patients, finger flexion delay was larger than in the healthy participant, while APAs timing actually showed a delay. However, while patient A produced the same intra-limb APA pattern observed in healthy individuals, patient B had an inverted intra-limb APA in BB, which underwent excitation instead of inhibition.

Figure 1 illustrates the EMG and kinematics recordings obtained in one representative healthy participant (HEALTHY) and two PD patients (PARKINSON A and B), who were representative of a normal APA pattern in the three recorded postural muscles and of an altered pattern in BB, respectively. Taking the onset of prime mover FDS EMG as time reference, the index-finger flexion occurred with a lower delay in the healthy participant (∼25 ms) than in both patients (∼70 ms in A and ∼40 in B). With regard to postural muscles, in the healthy participant the activation of FDS was preceded by an inhibitory intra-limb APA in BB and AD, whose activity was reduced with respect to the mean reference level, and almost simultaneous to an excitatory intra-limb APA in TB. Postural activities of similar sign, even if slightly delayed, could be observed also in the PD patient A, while patient B showed a change in pattern, as the intra-limb APA in BB muscle was reversed (excitatory instead of inhibitory).

### Kinematics Parameters

**FIGURE 2:**
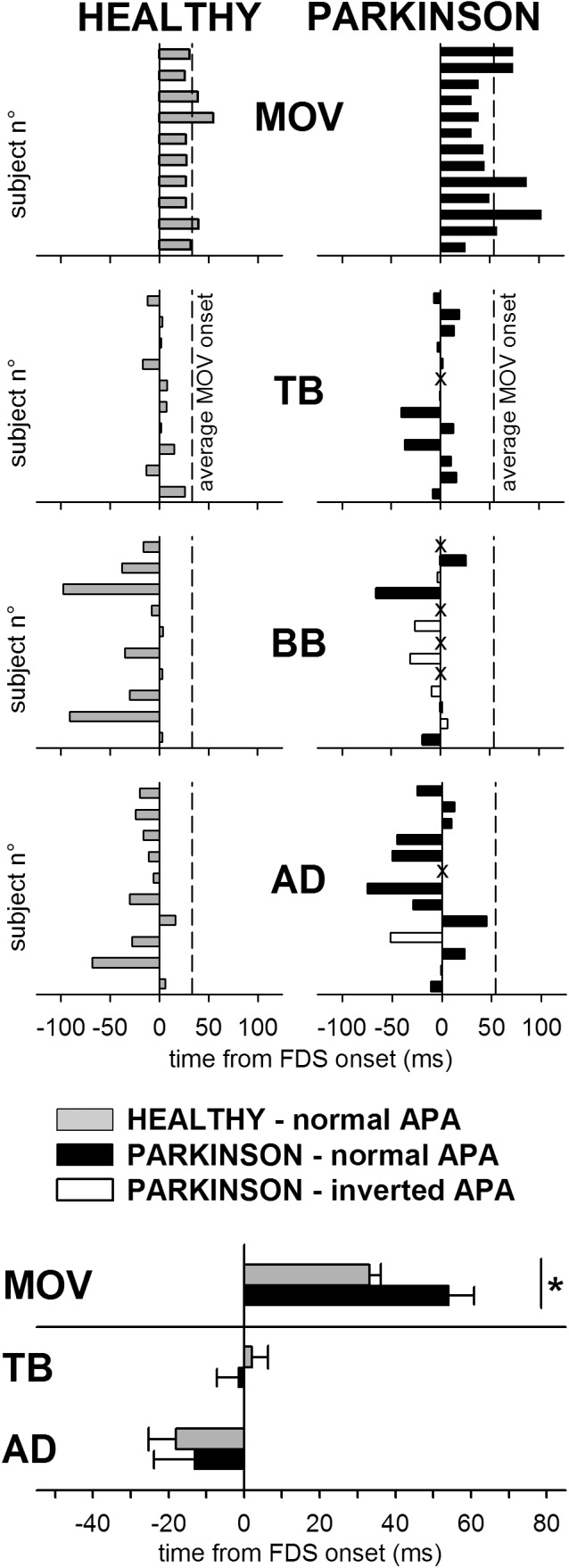
Comparison of the intra-limb APA chain in healthy subjects (HEALTHY) and PD patients (PARKINSON). Latencies of finger flexion (MOV) and intra-limb APAs in TB, BB, and AD are plotted with respect to onset of FDS. Each single participant is represented in the upper panels, where dashed lines mark the average movement latency for either group of participants. (For PD patients, latencies of intra-limb APAs which had the same sign observed in all healthy individuals (excitatory in TB and inhibitory in BB and AD) are plotted by black bars, latencies of intra-limb APAs of inverted sign are plotted by white bars and lack of intra-limb APAs is marked by “X.” Note that in several patients intra-limb APAs in BB were lacking or even inverted. The lowermost panel shows, for both groups, the mean latency (±SE) of the onset of finger flexion and of the intra-limb APAs in TB and AD, excluding data from those PD patients in which the APA was lacking or inverted. The asterisk marks the significant difference. The BB muscle was excluded from the latency comparison because only four patients had an APA of the same sign normally observed in healthy subjects.)

The individual latencies of index-finger flexion in HEALTHY and PARKINSON participants are illustrated in the upper panels of Figure 2, while inferential statistics are plotted in the lowermost panel. Compared to healthy participants, the latency of movement onset was larger in PD patients and showed a greater between-subjects variability. Statistical analysis confirmed such results, both with regard to mean values (*t*-test *t*_16.27_ = 2.911, *p* = 0.010) and to variability (Levene’s *F*_1,21_ = 8.677, *p* = 0.0077). Movement amplitude and duration are reported in Table 2, both as individual values and as mean ± SE. Also mean amplitude was significantly lower in PARKINSON vs. HEALTHY participants (*t*_21_ = 5.030, *p* < 0.0001), but with no significant differences in between-subjects variability (*F*_1,21_= 3.118, *p* = 0.0919). Movement duration, instead, was comparable both in mean value (*t*_21_ = 0.686, *p* < 0.5004) and variability (*F*_1,21_= 1.326, *p* = 0.2624).

### Intra-Limb APA Latency

The individual latencies of intra-limb APAs in HEALTHY and PARKINSON subjects are illustrated in the central panels of Figure 2. The fact that in all participants the identified APAs had a lower latency with respect to the index-finger movement witnesses the anticipatory nature of the postural muscles recruitment. Inferential statistics are plotted in the lowermost panel, showing that the latencies of intra-limb APAs in TB and AD muscles were comparable, both in mean value and variability, between the PARKINSON and HEALTHY groups. This was statistically confirmed by *t*-tests (for TB, *t*_20_ = 0.510, *p* = 0.6154; for AD, *t*_19_ = 0.372, *p* = 0.7137) and Levene’s tests (for TB, *F*_1,20_= 1.036, *p* = 0.3208; for AD, *F*_1,19_= 2.857, *p* = 0.1073). Note that the lowermost panel and the related statistical comparisons regarded data from all HEALTHY subjects (gray solid bars) vs. data from those PARKINSON patients which had an intra-limb APA of the same sign normally observed in HEALTHY subjects (black solid bars). Data from those patients in which the intra-limb APA was lacking (marked by an “X” in the central panels) or inverted (white bars) were excluded from the analyses. Because only four PD patients had an intra-limb APA of normal sign in BB, thus resulting in a very insufficient sample size, latency data from that muscle were excluded from statistics. For the same reason, it was not feasible to subdivide the latency comparison into different subgroups.

### Inter-Limb APA Pattern

The central panels of Figure 2 also illustrate that while all HEALTHY subjects presented intra-limb APAs of the same sign (excitatory in TB and inhibitory in BB and AD, gray bars), in some PD patients the intra-limb APA was lacking (“X”) or had the opposite sign with respect to what normally observed in HEALTHY subjects (white bars). While this seldom occurred for TB and AD (one patient lacked APAs in both muscles while another had an inverted APA in AD), it was not the case for BB. For this muscle, indeed, only 4 out of 13 patients had an inhibitory intra-limb APA, while 5 had an inverted APA and in 4 the APA was lacking. The Freeman-Halton extension of the non-parametric Fisher Exact test proved that the frequency of occurrence of *normal sign*, *inverted sign*, and *lacking* intra-limb BB APAs was significantly different (*p* = 0.0016) in the PARKINSON group (4, 5, and 4, respectively) vs. the HEALTHY one (10, 0, and 0).

### Secondary Measurements

For the sake of completeness, linear correlations were drawn between the *latency* of intra-limb APAs in TB or AD and the demographic and clinical parameters of PD patients, which are illustrated in Table 1. Non-parametric correlations were also drawn between the sign of intra-limb APAs in BB and those same parameters. Such correlations never reached significance (in all cases *p* > 0.28).

## Discussion

Present results show that the pattern of intra-limb APAs, that stabilize the arm when briskly flexing the index-finger (excitatory APA in TB and inhibitory in BB and AD), may be disrupted in PD patients, indirectly suggesting that basal ganglia could participate also in intra-limb postural control.

The pattern disruption, in particular the presence of an inverted intra-limb APA, mainly regarded the BB muscle, with sporadic occurrence in AD. One possible explanation is that the PD patients enrolled in this study, despite having received the first diagnosis from 3 to 12 years (Table 2), were in an initial stage of the disease, as witnessed by the moderate UPDRS-III scores. This could also justify the lack of significant correlations we observed between the intra-limb APA latencies or sign and the demographic and clinical parameters of PD patients. Another possible reason for the APA sign reversal occurring more frequently in BB than in TB and AD, could be the fact that if one approximates the whole arm as a rigid body, the reactive torque induced by the index-finger flexion should be the same on the elbow and the shoulder, because the lever-arm between the metacarpophalangeal joint and each of those two joints was identical (recall that the upper arm was vertical and hand prone in axis with the horizontal forearm). However, much more mass should be moved in order to flex the arm at the shoulder rather than at the elbow, so that it could be argued that the *TB-BB co-contraction* strategy adopted by some of the patients mainly aimed at increasing the elbow stiffness so as to discharge the perturbation on a larger sprung mass and, consequently, attenuate the unwanted displacement at the shoulder level. Moreover, with regard to focal movement kinematics, present data confirmed that PD patients were slower than age-matched healthy subjects, not only for what regards average speed but also in terms of prime mover recruitment, as witnessed by the longer delay between FDS activation and movement onset. However, such result should not have biased the observed intra-limb APA alteration because a previous study (Esposti et al., 2015) demonstrated that (i) intra-limb APAs are affected by the *intended* movement speed, not the actual one, and in this regard both healthy subjects and PD patients had to move at their fastest speed; (ii) even when moving at 50% of their fastest speed, healthy subjects did never show any reversal of the APA sign. Finally, the control experiments involved a cohort of healthy subjects of comparable age. This was chosen considering that APAs programming is affected by age both in self-initiated movements (Man’kovskii et al., 1980; Inglin and Woollacott, 1988; Rogers et al., 1992; Woollacott and Manchester, 1993) and when APAs are produced in order to respond to an external postural perturbation (Kanekar and Aruin, 2014).

The finding that the pattern of intra-limb APAs may be disrupted in some PD patients adds to the observations carried out in ataxic patients (Bruttini et al., 2015), which showed an altered intra-limb APA timing in absence of significant pattern disruptions. These results suggest a pathophysiological frame that well fits with the known roles of basal ganglia and cerebellum in selecting the correct motor program and temporizing the motor output, respectively (Grillner et al., 2005; Diedrichsen et al., 2007). In this regards, two recent results are also worth noting: first, literature reports proofs that basal ganglia and cerebellum are reciprocally interconnected through the pedunculopontine tegmental nucleus (see Wu and Hallett, 2013 for a review; Mori et al., 2016). The information exchange through these connections could justify the partial overlap observed between the symptomatic framework of cerebellum and basal-ganglia pathologies (Bostan and Strick, 2018). This has also been observed in intra-limb APAs, indeed Bruttini et al. (2015) reported cases of lacking intra-limb APAs in cerebellar ataxic patients, while signs of altered intra-limb APAs timing in parkinsonian patients are reported in the present paper (see Figure 1). Second, it has been reported that Parkinson’s disease, especially in its later phase, may also affect the cerebellum (Wu and Hallett, 2013). The same review paper also indicate that the cerebellum activation is abnormally high in PD patients performing various upper limb movements and hypothesize that at the initial stage of the disease the cerebello-thalamo-cortical loop may act so as to compensate for the progressive impairment of the striato-thalamo-cortical circuit (see also Blesa et al., 2007). Consequently, once the parkinsonian degeneration had affected the cerebellum, its compensation would fade-out, leading to a quicker development of the motor impairments.

While many observations confirmed that *intra-* and *inter-limb* APAs share so many behavioral properties that they are seemingly parts of the same phenomenon (Cavallari et al., 2016), it should be noted that our results only partially fit with data on APAs during gait initiation in PD patients. In the latter framework, indeed, some studies reported an altered APA pattern (e.g., Crenna and Frigo, 1991) while other studies reported delayed APAs in the absence of pattern disruptions (Delval et al., 2014). Such discrepancy could stem from the different mechanical context characterizing the two cases: (i) when the voluntary movement is limited to only part of the body, e.g., one or both arms, APAs *counteract the interaction forces* so as to grant that the rest of the body stands still; (ii) when the voluntary movement involves the whole body, like in gait, what are commonly called APAs are instead those actions that *produce the de-stabilizing forces* leading to the movement of the CoM (Jian et al., 1993; Elble et al., 1994; Lepers and Brenière, 1995; see Yiou et al., 2017 for a review). In particular, many studies about gait initiation considered APAs those co-ordinated activities in muscles acting on both ankles (tibialis anterior and gastrocnemius/soleus) that preceded the heel-off, taking the latter as the onset of the focal movement (Crenna et al., 2006; Honeine et al., 2016). However, those actions actually moved the Center of Pressure backward and toward the “future” leading foot, *directly producing* a shift of the CoM forward and toward the “future” trailing foot. Therefore, it might be even proposed that the forward shift of the CoM “*is*” the correct onset of gait, so that APAs should be searched for not before heel-off but before CoM displacement. If so, situations (i) and (ii), described above, appear to be conceptually different, so that a direct comparison of what is classically called APA in the two cases is not feasible.

## Author Contributions

PC and II conceived the study. II, NP,and UR-P recruited the patients and provided their clinical evaluation. FB, RE, and SM conducted the experiments and analyzed the results. PC, FB, and RE drafted the paper. All authors contributed to and approved the final version.

## Conflict of Interest Statement

The authors declare that the research was conducted in the absence of any commercial or financial relationships that could be construed as a potential conflict of interest.
